# Comparison of coronary CT angiography and invasive coronary angiography results

**DOI:** 10.1007/s11845-024-03745-y

**Published:** 2024-07-04

**Authors:** Muhammed Tekinhatun, İbrahim Akbudak, Mehmet Özbek, Mehmet Turmak

**Affiliations:** 1 Department of Radiology, Faculty of Medicine, Dicle University, Diyarbakir, Türkiye; 2 Department of Cardiology, Faculty of Medicine, Dicle University, Diyarbakir, Türkiye

**Keywords:** CAD-RADS, Coronary artery disease, Coronary computed tomography angiography, Non-invasive imaging techniques

## Abstract

**Introduction:**

Coronary artery disease (CAD) is a leading cause of death worldwide. Accurate diagnosis and management are critical. Non-invasive imaging, such as coronary computed tomography angiography (CCTA), is vital for early diagnosis and treatment planning. This study evaluates the accuracy of CAD-Reporting and Data System (CAD-RADS) scoring and the compatibility between CCTA and invasive coronary angiography (ICA) in patients suspected of having CAD.

**Materials and methods:**

From January 1, 2022 to January 15, 2024, 214 patients suspected of CAD underwent both CCTA and ICA. CCTA artifacts led to the exclusion of 32 patients and 128 vessels, leaving 586 vessels for analysis. CAD-RADS scoring categorized coronary stenosis. Diagnostic performance was measured by specificity, sensitivity, accuracy, positive and negative predictive value (NPV). Extracardiac findings were analyzed with a wide field of view (FOV) during CCTA.

**Results:**

A total of 214 patients (67.3% male, median age 56) were examined. Hypertension, smoking, calcium score, and high-risk plaques correlated with CCTA and ICA CAD-RADS scores; calcium score also related to hypertension, smoking, diabetes, and dyslipidemia (*p* < 0.05). CCTA showed a sensitivity of 80.8% and NPV of 90.3% for detecting stenosis of 70% or more; for 50% stenosis, sensitivity was 93.5% and NPV 92.1%. Agreement between CCTA and ICA was excellent in bypass patients; stenosis detection in stented patients had 85.7% sensitivity and 96.2% NPV.

**Conclusion:**

This study highlights the importance of CAD-RADS and CCTA in CAD diagnosis and treatment planning. CCTA effectively evaluates stents and grafts, emphasizing the benefits of extracardiac findings and a wide FOV.

## Introduction

Coronary artery disease (CAD) is one of the leading causes of death worldwide, despite all scientific advances [1]. Therefore, accurate diagnosis and effective planning are crucial. Electrocardiogram (ECG)-gated coronary computed tomography (CT) angiography, widely used to minimize the disadvantages of invasive imaging methods, plays a crucial role in the diagnosis of CAD. It aids in disease evaluation, early diagnosis and improvement of treatment processes [1].

In recent years, efforts have been made to establish a common communication language between cardiologists and radiologists through the Coronary Artery Disease Reporting and Data System (CAD-RADS) [2]. The main goals of CAD-RADS are to enhance communication between interpretive and guiding clinicians and to ensure better quality patients management through collaboration. In this system, severe stenosis is defined as 70% or more. Medical treatment and additional evaluation methods are recommended for patients with 50–69% stenosis, while invasive treatment methods are recommended for those with 70% and greater stenosis [2].

This study aims to evaluate the accuracy of the CAD-RADS scoring system, the compatibility between coronary computed tomography angiography (CCTA) and invasive coronary angiography (ICA), and the treatments applied by examining patients who were evaluated with CCTA and subsequently underwent ICA.

## Materials and methods

### Patient selection

The study was approved by the local ethics committee. A retrospective analysis was conducted on 2,120 patients who underwent CCTA in our radiology clinic for suspected CAD between January 1, 2022, and January 15, 2024. Patients examined with both CCTA and invasive coronary angiography (ICA) were included in the study (n: 246). Twenty-two patients who had a gap of more than one month between CCTA and ICA, and ten patients with artifact-laden coronary artery segments in CCTA images due to movement, cardiac arrhythmia, and respiratory artifacts (CAD-RADS N) were excluded from the study (128 vessels). Consequently, 214 patients and 586 vessels were analyzed.

### CCTA procedure

In our clinic, routine CCTA examinations are performed using a dual-source CT scanner with a 128 × 2-slice capability and two X-ray tubes positioned at a 95° angle (Somatom Definition Flash, Siemens Healthcare, Germany). Initially, non-contrast images with a slice thickness of 3 mm are obtained according to the Agatston classification used for calcium scoring (Ca score), using a standard prospective ECG-triggered protocol at 120 kVp. During this process, both the calcium score is determined and detailed images that encompass the entire thorax are obtained using a wide field of view (FOV). This also facilitates the acquisition of extracardiac findings. Subsequently, in the CCTA examination, the scanning area, starting from the level of the carina, extends to the diaphragm surface of the heart based on images taken for the Ca score. The contrast material is administered in a triphasic manner. Patients included in the study underwent these protocols and were evaluated using the electronic data system.

### ICA procedure

Invasive coronary angiography was performed using the transfemoral Judkins approach. During this procedure, the RCA and left main coronary artery branches were imaged in different projections. The cardiologist compared the stenoses in two different planes with a proximal normal segment and recorded the ratios.

### Image analysis

Images were transferred to a workstation (Syngo Via; Siemens Medical Solutions) for analysis. Initially, the calcium score was calculated. Extracardiac findings were evaluated. Detailed images were obtained from thin-section axial data using two-dimensional maximum intensity projection (MIP), multi-planar reconstruction (MPR), and three-dimensional volumetric imaging techniques. MPR and MIP images were utilized for a detailed examination of arterial lumens, arterial walls, and heart chambers.. Three-dimensional volumetric imaging was preferred for a clearer depiction of the complex anatomical structure of the coronary arteries and stenoses. The best systolic and diastolic reconstructions were automatically identified at the workstation, facilitating a detailed examination of the coronary arteries with minimal artifacts. All images were evaluated by consensus by two radiologists, each with five years of experience in cardiovascular radiology, who were blinded to the ICA images.

### CAD-RADS scoring

The assessment of coronary arteries utilized the CAD-RADS scoring criteria, categorizing based on the highest degree of stenosis observed in each patient. Stenosis classification was as follows: CAD-RADS 0 signifies no stenosis, CAD-RADS 1 indicates 0 to 24% stenosis, CAD-RADS 2 is 25 to 49% stenosis, CAD-RADS 3 ranges from 50 to 69% stenosis, CAD-RADS 4 entails 70% or more stenosis, and CAD-RADS 5 represents complete occlusion. Additional modifiers were applied to provide clarity; N for non-diagnostic, S for stent, G for graft, and HRP for high-risk plaque, defined by having at least two characteristics of HRP, including low attenuation plaque below 30 Hounsfield Units, positive remodeling, spotty calcification, or the “napkin ring” sign associated with low attenuation plaques [2].

LMCA, left anterior descending artery (LAD), left circumflex artery (LCX), and RCA were analyzed separately and similarly for vessel-based stenosis rates. Ca score was grouped as follows: 0, 1–100 = P1, 101–300 = P2, 301–999 = P3, > 999 = P4. Patients with a history of stent and bypass were not included in the Ca score evaluation. Treatments were classified as no treatment required, medical treatment, stent treatment, and surgery.

The diagnostic performances of CCTA and ICA were compared, and the effects of risk factors such as smoking, hypertension, dyslipidemia, and diabetes on Ca score and HRP diagnosis were examined. Additionally, non-contrast images covering the entire thorax with a wide FOV provided information about thoracic parenchyma, and triphasic scanning protocol provided insights into right heart chambers and pulmonary arteries. This allowed the evaluation of extracardiac and extracoronary cardiac findings.

### Statistical analysis

Statistical analyzes were performed using SPSS Statistic software 24 (SPSS Inc., Chicago, III). Categorical variables were presented as numbers and percentages. The sensitivity, specificity, positive predictive value (PPV), and negative predictive value (NPV) of detecting coronary stenosis by CTA were compared with ICA by using a 2 × 2 cross-tabulation model. To evaluate the association between variables, the Chi-square test or Fisher exact test was applied. *p* < 0.05 was considered statistically significant.

## Results

### Patients' basic characteristics

**Fig. 1:**
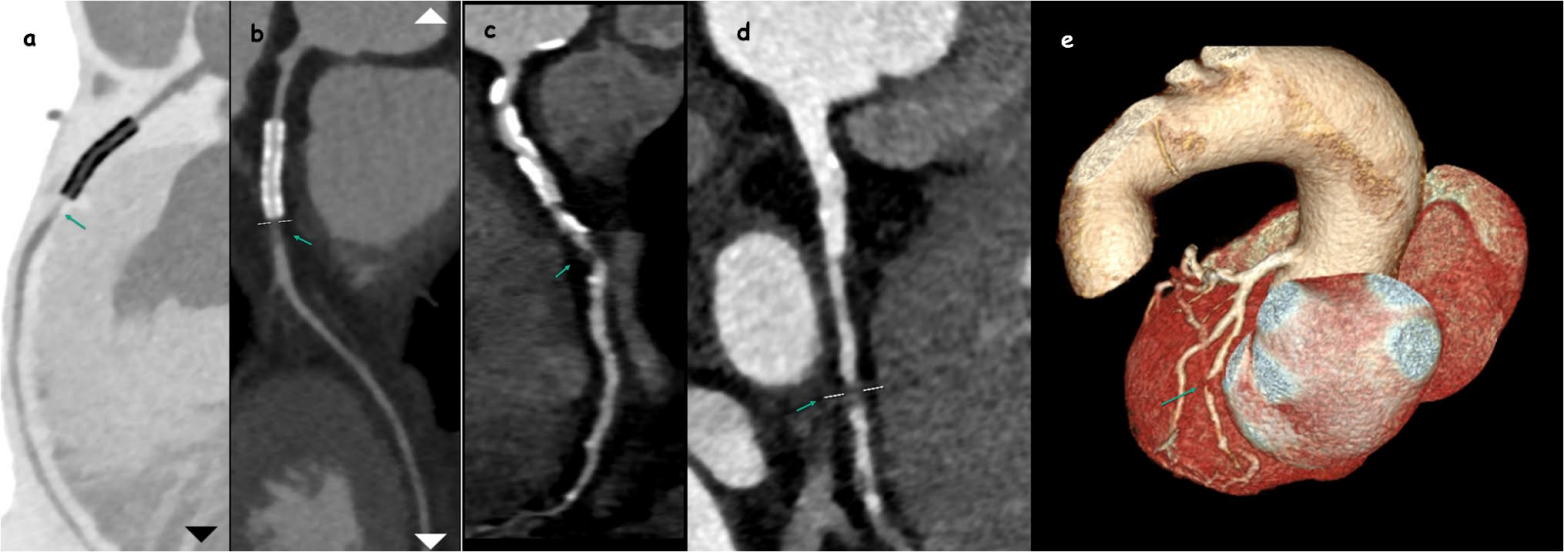
In the patient with a stent in the LAD, the stent is open but there is a CAD-RADS 4 lesion (arrow) (**a**: inverted image, **b**) in the distal part of the stent. In another patient, there is a CAD-RADS 4 lesion (arrow) in the LCX (**d**) and 3D image (**e**)

**Fig. 2:**
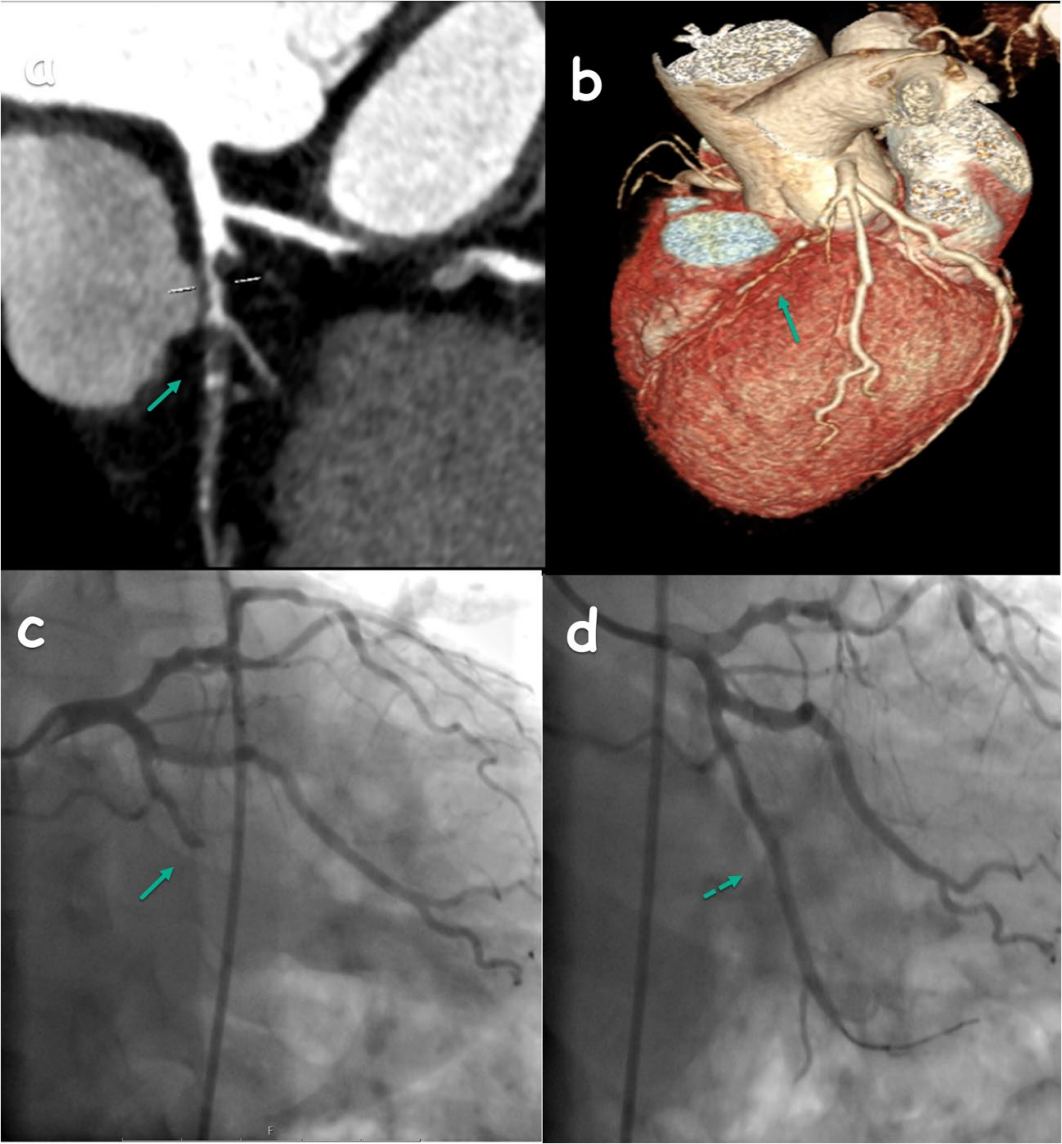
CCTA image (**a**, **b**: 3D image) and ICA image (**c**) of the patient with CAD-RADS 5 lesion (arrow) in the LCX show occlusion, and after the treatment the LCX is observed to be open (dashed arrow) (**d**)

**Fig. 3:**
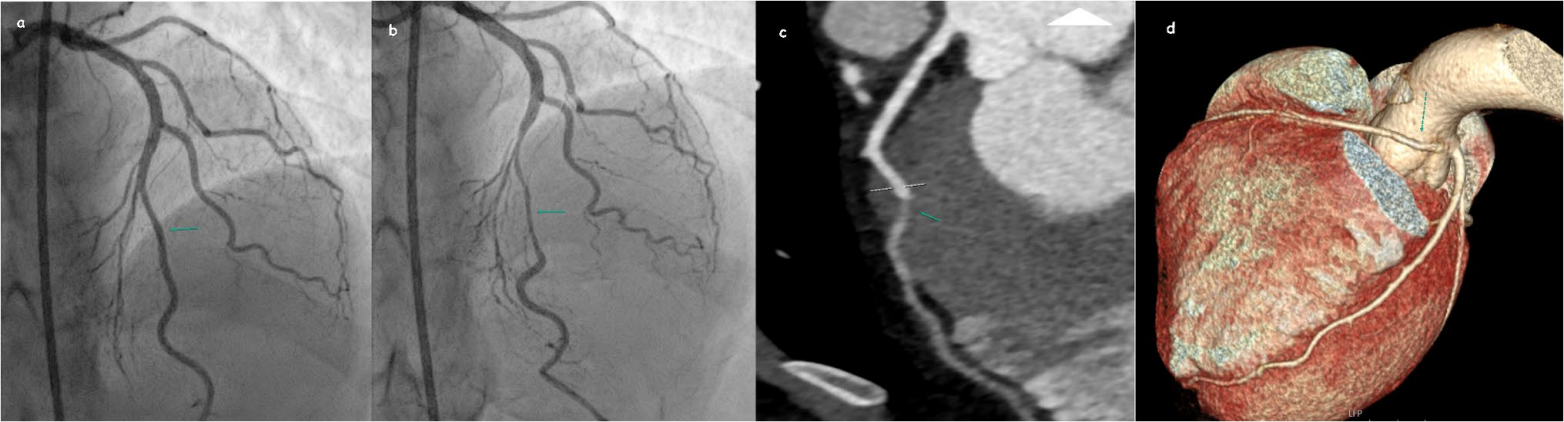
The image of the patient with a myocardial bridge during diastole (**a**) and systole (**b**) shows narrowing in the ICA (arrow), the CCTA image (**c**) of the patient shows a deep myocardial bridge (arrow), and another patient has a malignant interarterial course in which the RCA originates from the left coronary sinus (**d**) (dashed arrow)

In our study, 214 patients were evaluated, with 144 (67.3%) being male and 70 (32.7%) female. The median age was 56 years, ranging from 24 to 82. Detailed patient demographics are provided in Table 1. An analysis of Ca scoring revealed that 52 (24.3%) of the patients had a score of 0, 60 (28%) were scored between 1–100 (P1), 35 (16.4%) between 101–300 (P2), 30 (14%) between 301–1000 (P3), and 11 (5.1%) exceeded a score of 1000 (P4). Moreover, regarding the CAD-RADS evaluation, 18 (8.4%) patients were categorized as CAD-RADS 0, 42 (19.6%) as CAD-RADS 1, 46 (21.5%) as CAD-RADS 2, 35 (16.4%) as CAD-RADS 3, 57 (26.6%) as CAD-RADS 4 (Fig. 1), and 16 (7.5%) as CAD-RADS 5 (Fig. 2). Furthermore, 18 (8.4%) patients had a myocardial bridge (Fig. 3), and 32 (15%) had HRP. Of all the patients included in the study, 11 (5.1%) had one stent, 7 (3.3%) had two stents, 2 (0.9%) had three stents, and 1 (0.5%) had four stents. Additionally, 4 (1.9%) patients had two-vessel bypass grafts, and 4 (1.9%) had three-vessel bypass grafts.

**Table 1:** Demographic, Ca score, stent, and graft characteristics of patients

Characteristics	*n*	%
Gender		
Male	144	67.3
Female	70	32.7
Age	24–82	median 56
Diabetes	66	30.8
Hypertension	110	51.4
Smoking	120	56.1
Dyslipidemia	52	24.3
Ca score		
0	52	24.3
1–100	60	28
101–300	35	16.4
301–1000	30	14
> 1000	11	5.1
Stent and greft patient	26	12.1
Bridge	18	8.4
High-risk plaque	32	15
Stent		
One stent	11	5.1
Two stent	7	3.3
Three stent	2	0.9
Four stent	1	0.5
Graft		
Two vessel	4	1.9
Three vessel	4	1.9

In the analysis, hypertension and smoking were found to be associated with CAD-RADS scoring (*p* = 0.006 and *p* = 0.002, respectively). Diabetes mellitus and dyslipidemia were not associated with CAD-RADS. Hypertension, smoking, diabetes mellitus, and dyslipidemia were all statistically significantly associated with Ca scores (*p* = 0.009, 0.019, 0.010, and 0.004, respectively).

Extracardiac and extracoronary findings detected in the wide FOV images obtained for the Ca score and during CCTA are presented in Table 2.

**Table 2:** Extracardiac and extracoronary findings

Findings	*n*	%
Emphysema	30	14
Aortic dilation	5	2.3
Bronchiectasis	1	0.5
Pulmonary embolism	1	0.5
Caseous calcification	1	0.5
Different ostium of LAD and LCX	3	1.4
Pulmonary artery hypoplasia	1	0.5
Non-specific interstitial pneumonia	2	0.9
Usual interstitial pneumonia	1	0.5
Pericardial effusion	1	0.5
Fistula	5	2.3
Atrial septal defect	2	0.9
Ventricular septal defect	1	0.5
Interatrial septal aneurysm	4	1.9
Interventricular septal aneurysm	2	0.9
Partial anomalous pulmonary venous return	2	0.9
Retroaortic LCX	2	0.9
Malignant interarterial course (RCA originating from left coronary sinus)	3	1.4
High-origin RCA	1	0.5
Respiratory bronchiolitis	6	2.8
Left atrial pseudoaneurysm	1	0.5
Left atrial dilation	1	0.5
Right ventricular dilation	1	0.5
Pulmonary hypertension and Persistent left superior vena cava	1	0.5
Malignant nodule	2	0.9

### Vessel-based evaluation results

In our study, sensitivity, specificity, positive predictive value (PPV), and negative predictive value (NPV) were calculated for vessel-based and patient-based analyses according to the 70% and 50% stenosis threshold values based on CAD-RADS 2.0. The results of the coronary vessel analysis for 856 vessels and the patient-based analysis are presented in Table 3.

**Table 3:** Coronary, vessel-based, and patient-based analysis

Coronary vessel analysis					
Vessel	Sensitivity (%)	Specificity (%)	PPV (%)	NPV (%)	
LMCA 70%	50.00	100.00	100.00	98.58	
LMCA 50%	71.43	99.52	83.33	99.04	
LAD 70%	73.58	93.79	79.59	91.52	
LAD 50%	90.59	80.62	75.49	92.86	
LCX 70%	64.29	98.92	90.00	94.82	
LCX 50%	71.43	95.35	78.95	93.18	
RCA 70%	80.00	98.88	93.33	96.20	
RCA 50%	78.57	94.94	84.62	92.59	
Vessel-based analysis					
Stenosis rate (All vessels n:856)	Sensitivity (%)	Specificity (%)	PPV (%)	NPV (%)	Accuracy (%)
70% and above	72.1	98.1	86.3	95.5	88.3
50% and above	82.1	93.7	78.8	94.8	85.5
Patient-based analysis					
Stenosis rate	Sensitivity (%)	Specificity (%)	PPV (%)	NPV (%)	Accuracy (%)
70% and above	80.8	92.2	84.3	90.3	88.3
50% and above	93.5	77.4	80.8	92.1	85.5

The results demonstrate that CCTA, especially in patients with high Ca scores, can detect stenoses with high sensitivity and accuracy. However, there are diagnostic challenges in patients with Ca scores P2 and P3, which have led to overdiagnosis (Table 4).

**Table 4:** Ca score analysis

Ca score	Sensitivity (%50)	Specificity (%50)	PPV (%50)	NPV (%50)	Sensitivity (%70)	Specificity (%70)	PPV (%70)	NPV (%70)
0	87.5	93.6	70	97.8	83.3	100	100	98
1	83.3	82.9	80.6	85.3	73.9	92.9	85	86.7
2	100	30.8	71.8	100	76.9	87	76.9	87
3	100	14.3	80	100	82.4	78.6	82.4	78.6
4	100	100	100	100	100	66.7	71.4	100

### HRP and Ca score analysis

**Fig. 4:**
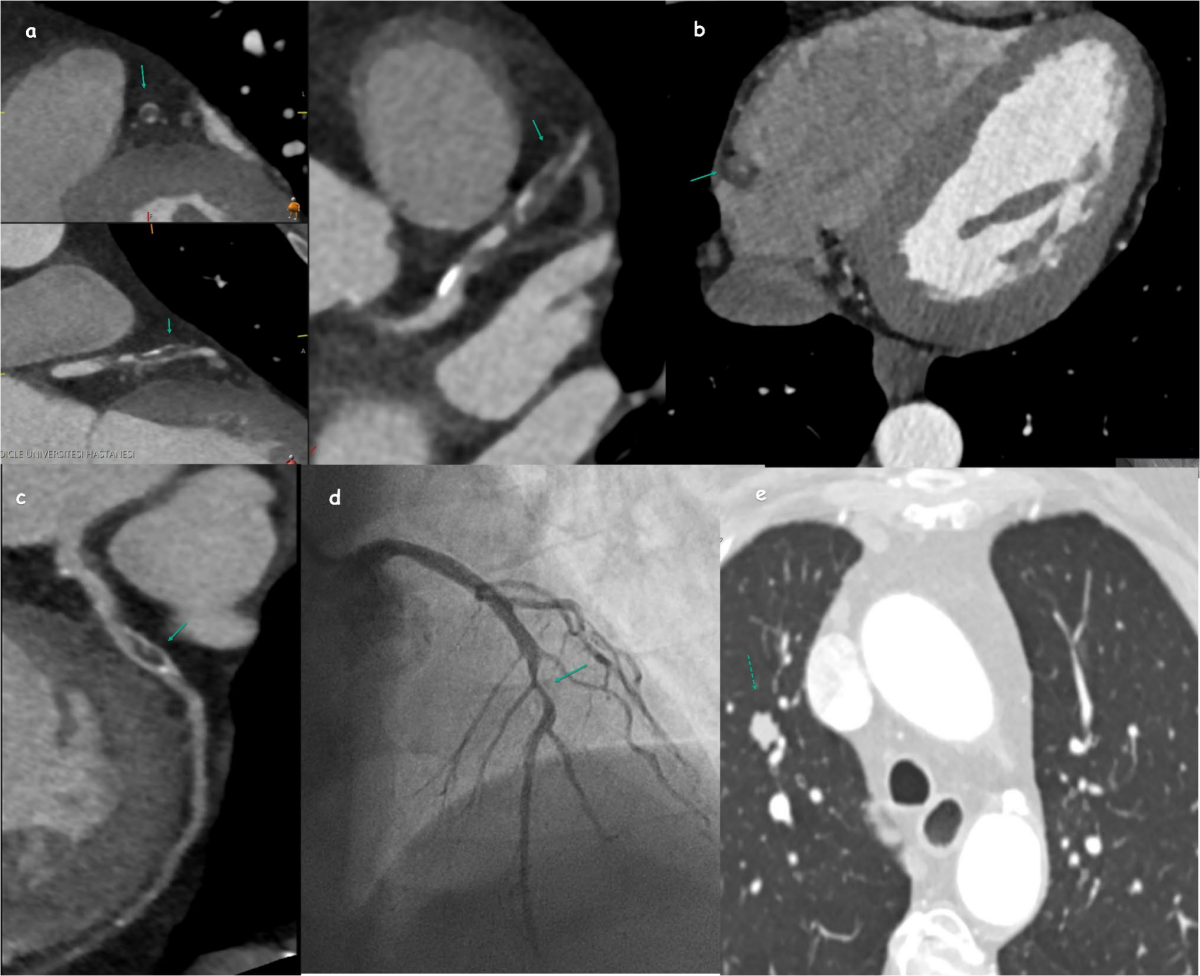
In the patient with CAD-RADS 4 lesion in the LAD; In coronal, sagittal and axial images (**a**), low-density HRP giving a napkin ring appearance (arrow), HRP showing positive remodeling in the RCA (**b**), CCTA (**c**) and ICA image of low-density HRP causing CAD-RADS 3 lesions (**d**) (arrow), nodule with irregular borders detected in the parenchymal window (dashed arrow) (**e**)

Our study found that 32 patients (15%) had HRP (Fig. 4), and a significant relationship was found between vulnerable plaque and Ca score (*p* = 0.021). Additionally, HRP was significantly associated with both CCTA CAD-RADS score and ICA CAD-RADS score (*p* < 0.05). The median age of patients with vulnerable plaques was 56 (range: 35–73).

### Bypass surgery analysis

In our study, 8 patients (3.7%) had bypass surgery, with a total of 20 grafts. The agreement between CCTA and ICA was excellent in patients with grafts, with 14 patients (70%) having stenosis. Sensitivity, specificity, PPV, and NPV were all 100%.

### Stent analysis

**Fig. 5:**
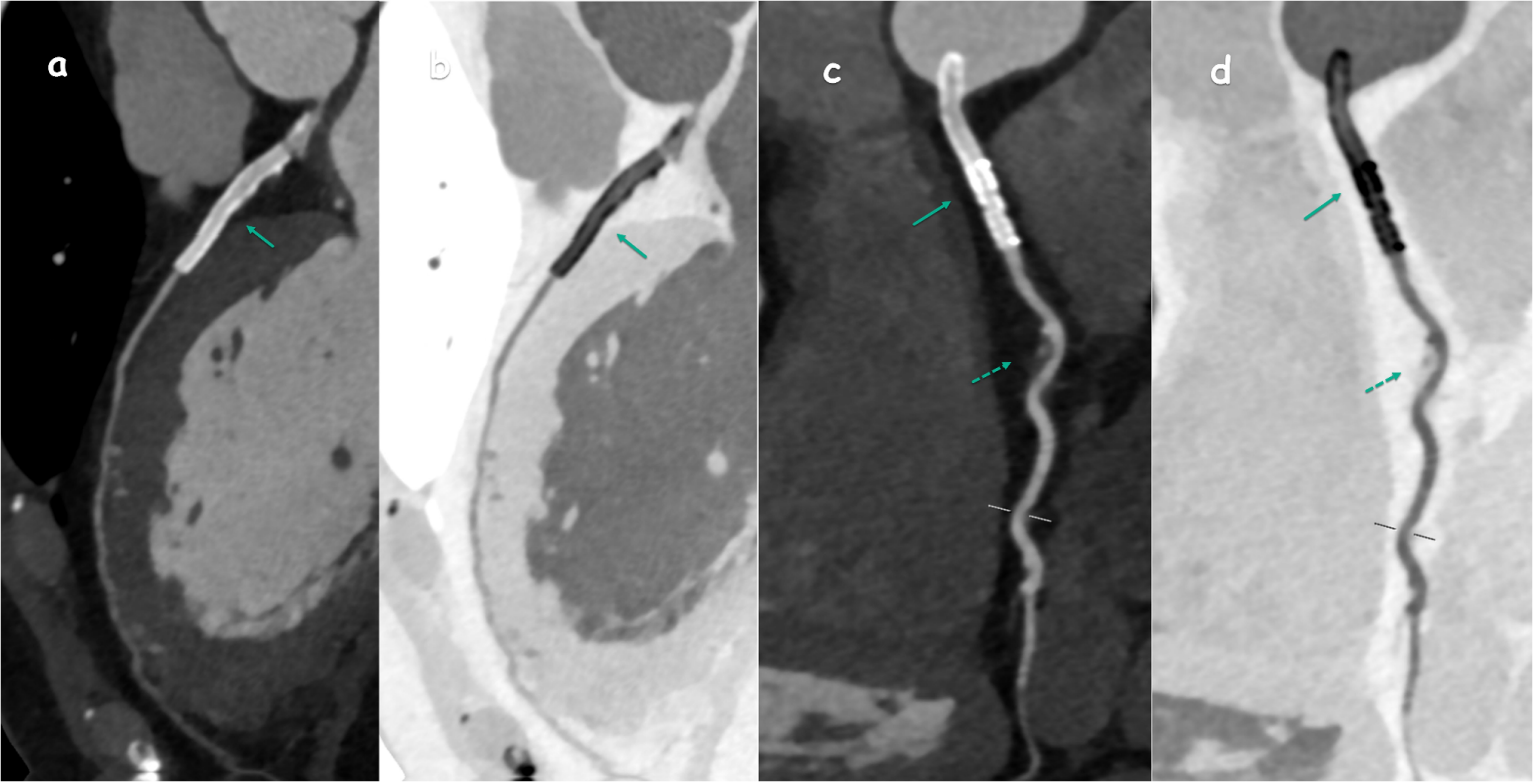
In patients with stents, the stents are seen to be open (arrow) (**a**, **b**), in another patient with stents, a mixed type plaque causing 40% stenosis is also seen (**c**, **d**) (CAD-RADS 2)

In our study, 21 patients (9.8%) had stents, with a total of 35 stents (Fig. 5). Seven patients (20%) had stenosis in stented vessels. Sensitivity was 85.7%, specificity was 89.3%, PPV was 66.7%, and NPV was 96.2%.

### CAD-RADS 3 analysis

We had 35 patients (16.4%) with CAD-RADS 3, of whom 31 (88.6%) were treated medically (median age: 59, male ratio: 61.3%), while 4 (11.4%) received stent treatment (median age: 64, male ratio: 75%). These patients were older and predominantly male, but the differences were not statistically significant. There were no significant differences in smoking, diabetes, hypertension, or dyslipidemia. All CAD-RADS 3 patients were monitored for major cardiac events; no major cardiac events were observed in the 31 medically treated patients (minimum 3 months, maximum 22 months, average 10 months) or in the 4 stent-treated patients (minimum 4 months, maximum 8 months, average 6 months).

## Discussion

This study focuses on evaluating the compatibility between CCTA and ICA using CAD-RADS 2.0 [2] in the diagnosis of CAD. This study, conducted with a large patient group, concentrates on the differences in diagnostic performance between CCTA and ICA, the impact of the Ca score, and the significance of the presence of HRP, offering an innovative perspective on CAD diagnosis and treatment approaches.

CAD continues to be a significant health issue in both developed and developing countries and maintains its position among the leading causes of death despite advancements in diagnostic methods and treatment options [3, 4]. In our study, with 144 (67.3%) being male and 70 (32.7%) female. The median age of the patients was 56 (range: 24–82), similar to studies in the literature indicating that CAD is more common in males and at advanced ages [5–7].

According to CAD-RADS 2.0, Ca scores are classified as P1, P2, P3, and P4 [2]. Our study found a statistically significant association between the Ca score and CAD-RADS score, consistent with other studies on Ca scoringn [8–13]. Specifically, CCTA provides detailed information about the characterization of the vessel wall and plaques, offering advantages over ICA. It suggests that CCTA may be a more useful guide for clinicians in follow-up and treatment.

When comparing our findings with a study conducted in 2024 [14], some differences are observed. In our study, the proportion of participants with a zero coronary Ca score is lower at 24.3%, and non-zero Ca scores appear approximately 15 years earlier in men than in women. Additionally, high-risk categorization (75th percentile) for Ca scores is reached at the age of 37 for men and 41 for women, indicating an earlier onset compared to the age limits set by the previous study. These differences can be attributed to our study population, which includes individuals with a higher suspicion of CAD, undergoing both CCTA and ICA; this suggests that our findings are derived from a high-risk group and should be evaluated in this context.

Our study includes patients with stents and grafts. Similar diagnostic parameters were identified between CCTA and ICA in evaluating these patients. Particularly, CCTA has been shown to offer comparable results to ICA in assessing stent and graft lumens. Additionally, CCTA provides extra advantages in evaluating extracardiac findings, extracoronary findings, and the assessment of intimal walls and lesion locations [15–21].

Our study includes the evaluation of extracardiac findings (Table 2) as recommended by the most latest version of CAD-RADS [2]. The capability of CCTA to diagnose these findings presents a significant advantage over ICA. Particularly when cardiac findings do not explain the patient's symptoms, exploring alternative causes is essential for appropriate treatment. Potential other causes of chest pain include smoking-related lung diseases, interstitial lung diseases, malignancies, anatomical anomalies, and pulmonary embolism. These conditions can be easily detected especially with the non-contrast CT examination for Ca scoring and the triphasic scanning protocol of CCTA [22–25]. Given the diverse patient population encountered in university hospitals, such examinations are deemed essential. Therefore, the examination for Ca scoring was conducted with a wide FOV covering the entire thorax and using a triphasic CCTA scanning protocol. In the literature, there are limited studies advocating the use of a wide FOV instead of a cardiac-focused FOV for diagnostic and cost benefits [24–26]. Therefore, especially for patients at medium and high risk recommended for CCTA, we suggest that the images taken for Ca scoring be scanned with a wide FOV considering factors such as age and smoking habits. According to the new guidelines published by the American Cancer Society in 2023, annual lung cancer screening is recommended for individuals aged 50 to 80 who currently smoke or have quit in the past and have a history of 20 pack-years or more. This recommendation can be combined with the recommended screenings for Ca scoring, making it possible to conduct both screenings simultaneously [27].

Studies have examined with similar rates in the literature (In patient-based analysis, patients with CAD-RADS 3 and higher scores) have shown that CCTA successfully detects [28–33] CAD with high sensitivity and specificity rates. Also, the study by Nikolou and colleagues [34] has shown high diagnostic performance for stenoses of 75% and greater. CCTA's diagnostic performance, when compared to the reference standard ICA, has been indicated to show high sensitivity and specificity in meta-analyses with exercise ECG and single photon emission computed tomography (SPECT) [35–37]. A study published in 2024 has demonstrated significant diagnostic performance of CCTA in detecting significant stenoses (more than 50%) with high sensitivity, specificity, NPV, and PPV [38]. All these studies and our research emphasize the strong diagnostic capacity of CCTA in the diagnosis of CAD and its advantages compared to alternative diagnostic methods.

In graft evaluation, while ICA is considered the gold standard, it increases the radiation dose exposed to both the patient and the doctor. Sometimes these evaluations can be inadequate [38, 39]. In a study conducted with 100 patients with bypass using ICA and CCTA, similar to our study, all diagnostic performance parameters were obtained as 100% [40]. In another study conducted on 84 patients with grafts, sensitivity was reported as 97% and specificity as 100% [41]. A meta-analysis examining 12 studies evaluating patients with grafts found sensitivity and specificity of 98% [42]. In our study, there were 20 grafts, and the evaluation in patients with grafts found perfect agreement between CCTA and ICA, with sensitivity, specificity, PPV, and NPV all found to be 100%.

In our study, there are a total of 35 stents in 21 patients, with CCTA finding sensitivity of 85.7%, specificity of 89.3%, PPV of 66.7%, and NPV of 96.2%. A meta-analysis evaluating 35 studies on patients with stents found sensitivity of 90% and specificity of 94% for stenoses of 50% and greater [43].

In the literature, the presence of HRP, even in low-risk patients such as those with CAD-RADS 1 and 2, is suggested to require more aggressive preventive treatments. In the literature, CT Fractional Flow Reserve (CT FFR), CT perfusion (CTP), stress imaging or, if necessary, ICA are recommended as additional examination in patients with CAD-RADS 3 and HRP [2]. The presence of HRP is thought to triple a patient's risk of death or nonfatal myocardial infarction (MI) [11]. A study found that HRP was less common in women and the risk of myocardial infarction (MI) was lower. Additionally, HRP was found to increase the risk of MI, independent of Ca score (Ca score), obstructive disease, gender and cardiovascular risk factors (Odds Ratio: 1,6) [44]. A 2024 article [45] emphasized that the optimal management of HRP has not yet been determined and that this issue needs further discussion. In our study, we found that HRP is more common in men and the average age is consistent with the literature. 13 patients with HRP (5 patients with CAD-RADS 1 and 2) received medical treatment, and 19 patients received stent and surgical intervention (11 stents, 8 surgeries). It is believed that the treatment approaches and follow-up of these patients could make significant contributions to the literature.

In the literature, it is recommended to use functional evaluation methods such as CT-FFR, CTP, or stress tests to document or rule out the presence of ischemia in patients with CAD-RADS 3 scores [2]. Our study emphasizes the need for personalized treatment for CAD-RADS 3 patients, as stated in the literature [2]. This approach helps determine the necessity of invasive treatment. In our study, CADRADS 3 patients were questioned for major cardiac events. A major cardiac event was not observed in 31 patients who received medical treatment (minimum 3 months, maximum 22 months, average 10 months) or four patients who received stent treatment (minimum 4 months, maximum 8 months, average 6 months). This finding is important, and similar follow-up studies with larger numbers of CAD-RADS 3 patients are needed.

CCTA has important advantages such as identifying complications related to stents and grafts, recognizing non-atherosclerotic stenosis, and determining plaque burden and characterization. These features make CCTA a valuable imaging method for both doctors and patients.

Our study is generally compatible with the treatment recommendations stated in the CAD-RADS score. The presence of HRP, which can be easily evaluated by CCTA, may increase the severity and possible risks of CAD. This supports the recommendation of more aggressive treatment methods, such as invasive evaluation and potential revascularization, especially for patients with CAD-RADS scores of 3, 4 and 5. The detection of HRP plays an important role in the early and accurate identification of CAD and is critical in determining appropriate treatment strategies.

Our study offers a significant advantage in evaluating patients with CAD-RADS 2.0 and presenting a high number of patients compared to similar studies in the literature. However, our study has some limitations. Particularly, the fact that the study is single-center and retrospective is an important limitation. Other limitations include the low number of patients with HRP and CAD-RADS 3 scores and the lack of advanced methods such as CT-FFR in our unit. Especially, we believe that diagnosing, treating, and following up patients with HRP and CAD-RADS 3 scores with more extensive participation studies is necessary for managing these patients..

In conclusion, our study supports the importance of HRP in CAD management and the efficacy of CCTA in detecting these plaques, while also demonstrating compliance with the treatment recommendations based on the CAD-RADS score. Additionally, our study emphasizes the advantages of the imaging protocol used for Ca scoring, taken with a wide FOV and a triphasic scanning protocol. These findings provide significant contributions to the development of diagnosis and treatment strategies for CAD.

## Data Availability

The data that support the findings of this study are available upon reasonable request.

## References

[1] Virani SS, Newby LK, Arnold SV, et al (2023) 2023 AHA/ACC/ACCP/ASPC/NLA/PCNA guideline for the management of patients with chronic coronary disease: a report of the American Heart Association/American College of Cardiology Joint Committee on Clinical Practice Guidelines. Circulation 148:e9–e119. 10.1161/CIR.0000000000001168 37471501 10.1161/CIR.0000000000001168

[2] Cury RC, Leipsic J, Abbara S, et al (2022) CAD-RADS^TM^ 2.0 - 2022 Coronary Artery Disease - Reporting and Data System An Expert Consensus Document of the Society of Cardiovascular Computed Tomography (SCCT), the American College of Cardiology (ACC), the American College of Radiology (ACR) and the N. Radiol Cardiothorac Imaging 4:e220183. 10.1148/ryct.220183 36339062 10.1148/ryct.220183 PMC9627235

[3] Nowbar AN, Gitto M, Howard JP, et al (2019) Mortality from ischemic heart disease. Circ Cardiovasc Qual Outcomes 12:e005375. 10.1161/CIRCOUTCOMES.118.005375 31163980 10.1161/CIRCOUTCOMES.118.005375 PMC6613716

[4] Chen X, Liu H-X, Yu X-Q, et al (2021) Standard modifiable cardiovascular risk factors and prognosis of acute coronary syndrome in younger patients. J Coll Physicians Surg Pak 31:1394–1398. 10.29271/jcpsp.2021.12.1394 34794276 10.29271/jcpsp.2021.12.1394

[5] Ekladious MEY, Guirguis MS, Haggag AM, Abdelrahman AS (2022) An Egyptian study to assess the accuracy and reliability of CAD-RADS CT coronary angiography algorithm in the evaluation of coronary artery disease. Egypt J Radiol Nucl Med 53:32. 10.1186/s43055-022-00705-3

[6] Mohammad AM, Rashad HH, Habeeb QS, et al (2021) Demographic, clinical and angiographic profile of coronary artery disease in kurdistan region of Iraq. Am J Cardiovasc Dis 11:39–45 33815918 PMC8012293

[7] Ahmadzadeh K, RoshdiDizaji S, Kiah M, et al (2023) The value of Coronary Artery Disease - Reporting and Data System (CAD-RADS) in outcome prediction of CAD patients; a systematic review and meta-analysis. Arch Acad Emerg Med 11:e45. 10.22037/aaem.v11i1.1997 37609531 10.22037/aaem.v11i1.1997 PMC10440753

[8] Shaw LJ, Blankstein R, Bax JJ, et al (2021) Society of cardiovascular computed tomography / North American society of cardiovascular imaging - expert consensus document on coronary CT imaging of atherosclerotic plaque. J Cardiovasc Comput Tomogr 15:93–109. 10.1016/j.jcct.2020.11.002 33303383 10.1016/j.jcct.2020.11.002

[9] Adamson PD, Williams MC, Dweck MR, et al (2019) Guiding therapy by coronary CT angiography improves outcomes in patients with stable chest pain. J Am Coll Cardiol 74:2058–2070. 10.1016/j.jacc.2019.07.085 31623764 10.1016/j.jacc.2019.07.085 PMC6899446

[10] Bittencourt MS, Hulten E, Ghoshhajra B, et al (2014) Prognostic value of nonobstructive and obstructive coronary artery disease detected by coronary computed tomography angiography to identify cardiovascular events. Circ Cardiovasc Imaging 7:282–291. 10.1161/CIRCIMAGING.113.001047 24550435 10.1161/CIRCIMAGING.113.001047

[11] Williams MC, Moss AJ, Dweck M, et al (2019) Coronary artery plaque characteristics associated with adverse outcomes in the SCOT-HEART study. J Am Coll Cardiol 73:291–301. 10.1016/j.jacc.2018.10.066 30678759 10.1016/j.jacc.2018.10.066 PMC6342893

[12] Mortensen MB, Dzaye O, Steffensen FH, et al (2020) Impact of plaque burden versus stenosis on ischemic events in patients with coronary atherosclerosis. J Am Coll Cardiol 76:2803–2813. 10.1016/j.jacc.2020.10.021 33303068 10.1016/j.jacc.2020.10.021

[13] Lin FY, Shaw LJ, Dunning AM, et al (2011) Mortality risk in symptomatic patients with nonobstructive coronary artery disease: a prospective 2-center study of 2,583 patients undergoing 64-detector row coronary computed tomographic angiography. J Am Coll Cardiol 58:510–519. 10.1016/j.jacc.2010.11.078 21777749 10.1016/j.jacc.2010.11.078

[14] Kılıçkap G, Tekdemir H, Bahadır K, et al (2024) Coronary artery calcium score percentiles: data from a single center in Turkey. Diagn Interv Radiol 30:21–27. 10.4274/dir.2023.232196 37317830 10.4274/dir.2023.232196 PMC10773180

[15] Alani A, Nakanishi R, Budoff MJ (2014) Recent ımprovement in coronary computed tomography angiography diagnostic accuracy. Clin Cardiol 37:428–433. 10.1002/clc.22286 24756932 10.1002/clc.22286 PMC6649438

[16] Zhao J, Zheng L, Yang Y (2011) Evaluation of coronary artery in-stent patency using 64-slice computed tomography. Coron Artery Dis 22(8):540–552. 10.1097/MCA.0b013e32834c1a28 21959714 10.1097/MCA.0b013e32834c1a28

[17] Budoff MJ, Dowe D, Jollis JG, et al (2008) Diagnostic performance of 64-multidetector row coronary computed tomographic angiography for evaluation of coronary artery stenosis in ındividuals without known coronary artery disease: results from the prospective multicenter ACCURACY (Assessment by Coro. J Am Coll Cardiol 52:1724–1732. 10.1016/j.jacc.2008.07.031 19007693 10.1016/j.jacc.2008.07.031

[18] Malagutti P, Nieman K, Meijboom WB, et al (2007) Use of 64-slice CT in symptomatic patients after coronary bypass surgery: evaluation of grafts and coronary arteries. Eur Heart J 28:1879–1885. 10.1093/eurheartj/ehl155 16847009 10.1093/eurheartj/ehl155

[19] Ropers D, Pohle F-K, Kuettner A, et al (2006) Diagnostic accuracy of noninvasive coronary angiography in patients after bypass surgery using 64-slice spiral computed tomography with 330-ms gantry rotation. Circulation 114:2334–2341. 10.1161/CIRCULATIONAHA.106.631051 17088461 10.1161/CIRCULATIONAHA.106.631051

[20] Chan M, Ridley L, Dunn DJ, et al (2016) A systematic review and meta-analysis of multidetector computed tomography in the assessment of coronary artery bypass grafts. Int J Cardiol 221:898–905. 10.1016/j.ijcard.2016.06.264 27439070 10.1016/j.ijcard.2016.06.264

[21] Machida H, Tanaka I, Fukui R, et al (2015) Current and novel ımaging techniques in coronary CT. Radiogr a Rev Publ Radiol Soc North Am Inc 35:991–1010. 10.1148/rg.2015140181 10.1148/rg.2015140181 26046942

[22] Johnson KM, Dennis JM, Dowe DA (2010) Extracardiac findings on coronary CT angiograms: limited versus complete image review. Am J Roentgenol 195:143–148. 10.2214/AJR.08.1050 20566808 10.2214/AJR.08.1050

[23] Teague SD, Rissing S, Mahenthiran J, Achenbach S (2012) Learning to interpret the extracardiac findings on coronary CT angiography examinations. J Cardiovasc Comput Tomogr 6:232–245. 10.1016/j.jcct.2012.02.007 22732196 10.1016/j.jcct.2012.02.007

[24] Kelion A, Sabharwal N, Holdsworth D, et al (2022) Clinical and economic impact of extracardiac lesions on coronary CT angiography. Heart 108:1461–1466. 10.1136/heartjnl-2021-320698 35318255 10.1136/heartjnl-2021-320698

[25] Otto CM (2022) Heartbeat: management of extracardiac findings on coronary computed tomographic angiography. Heart 108:1421–1423. 10.1136/heartjnl-2022-321725 36007933 10.1136/heartjnl-2022-321725

[26] Kim TJ, Han DH, Jin KN, Won Lee K (2010) Lung cancer detected at cardiac CT: prevalence, clinicoradiologic features, and importance of full-field-of-view images. Radiology 255:369–376. 10.1148/radiol.10091083 20413751 10.1148/radiol.10091083

[27] Wolf AMD, Oeffinger KC, Shih TY-C, et al (2024) Screening for lung cancer: 2023 guideline update from the American Cancer Society. CA Cancer J Clin 74:50–81. 10.3322/caac.21811 37909877 10.3322/caac.21811

[28] Madhok R, Aggarwal A (2014) Comparison of 128-slice dual source CT coronary angiography with ınvasive coronary angiography. J Clin Diagn Res 8:RC08-11. 10.7860/JCDR/2014/9568.4514 25121042 10.7860/JCDR/2014/9568.4514 PMC4129286

[29] Singh V, Kottapalli S, Gupta R, et al (2021) Diagnostic accuracy of 128-slice dual source CT coronary angiography with ınvasive catheter coronary angiography in a tertiary care teaching hospital. Pakistan J Med Heal Sci 15:2057–2062. 10.53350/pjmhs211562057

[30] Raff GL, Gallagher MJ, O’Neill WW, Goldstein JA (2005) Diagnostic accuracy of noninvasive coronary angiography using 64-slice spiral computed tomography. J Am Coll Cardiol 46:552–557. 10.1016/j.jacc.2005.05.056 16053973 10.1016/j.jacc.2005.05.056

[31] Neefjes LA, Rossi A, Genders TSS, et al (2013) Diagnostic accuracy of 128-slice dual-source CT coronary angiography: a randomized comparison of different acquisition protocols. Eur Radiol 23:614–622. 10.1007/s00330-012-2663-3 23052644 10.1007/s00330-012-2663-3

[32] Mander GTW, Dobeli K, Steffensen C, Munn Z (2021) Diagnostic accuracy of prospectively gated, 128-slice or greater CTCA at high heart rates: a systematic review and meta-analysis. J Med Radiat Sci 68:435–445. 10.1002/jmrs.525 34235885 10.1002/jmrs.525 PMC8656183

[33] Mühlenbruch G, Seyfarth T, Soo CS, et al (2007) Diagnostic value of 64-slice multi-detector row cardiac CTA in symptomatic patients. Eur Radiol 17:603–609. 10.1007/s00330-006-0429-5 17008986 10.1007/s00330-006-0429-5

[34] Nikolaou K, Knez A, Rist C, et al (2006) Accuracy of 64-MDCT in the diagnosis of ischemic heart disease. AJR Am J Roentgenol 187:111–117. 10.2214/AJR.05.1697 16794164 10.2214/AJR.05.1697

[35] Nielsen LH, Ortner N, Nørgaard BL, et al (2014) The diagnostic accuracy and outcomes after coronary computed tomography angiography vs. conventional functional testing in patients with stable angina pectoris: a systematic review and meta-analysis. Eur Hear J Cardiovasc Imaging 15:961–971. 10.1093/ehjci/jeu027 10.1093/ehjci/jeu027 24618659

[36] Yoshida K, Tanabe Y, Hosokawa T, et al (2024) Coronary computed tomography angiography for clinical practice. Jpn J Radiol. 10.1007/s11604-024-01543-1 38453814 10.1007/s11604-024-01543-1 PMC11139719

[37] Knuuti J, Ballo H, Juarez-Orozco LE, et al (2018) The performance of non-invasive tests to rule-in and rule-out significant coronary artery stenosis in patients with stable angina: a meta-analysis focused on post-test disease probability. Eur Heart J 39:3322–3330. 10.1093/eurheartj/ehy267 29850808 10.1093/eurheartj/ehy267

[38] Yan C, Liu J, Min J, et al (2024) Radiation dose and image quality of coronary CT angiography performed with whole-heart coverage CT scanner with 0.25s rotation time in patients with irregular heart rhythm. Heliyon 10:e25320. 10.1016/j.heliyon.2024.e25320 38375311 10.1016/j.heliyon.2024.e25320 PMC10875372

[39] Pesenti-Rossi D, Baron N, Georges J-L, et al (2014) Assessment of coronary bypass graft patency by first-line multi-detector computed tomography. Ann Cardiol Angeiol (Paris) 63:284–292. 10.1016/j.ancard.2014.08.011 25258019 10.1016/j.ancard.2014.08.011

[40] Mushtaq S, Conte E, Pontone G, et al (2020) Interpretability of coronary CT angiography performed with a novel whole-heart coverage high-definition CT scanner in 300 consecutive patients with coronary artery bypass grafts. J Cardiovasc Comput Tomogr 14:137–143. 10.1016/j.jcct.2019.08.004 31405817 10.1016/j.jcct.2019.08.004

[41] Martuscelli E, Romagnoli A, D’Eliseo A, et al (2004) Evaluation of venous and arterial conduit patency by 16-slice spiral computed tomography. Circulation 110:3234–3238. 10.1161/01.CIR.0000147277.52036.07 15533862 10.1161/01.CIR.0000147277.52036.07

[42] Barbero U, Iannaccone M, d’Ascenzo F, et al (2016) 64 slice-coronary computed tomography sensitivity and specificity in the evaluation of coronary artery bypass graft stenosis: a meta-analysis. Int J Cardiol 216:52–57. 10.1016/j.ijcard.2016.04.156 27140337 10.1016/j.ijcard.2016.04.156

[43] Dai T, Wang J-R, Hu P-F (2018) Diagnostic performance of computed tomography angiography in the detection of coronary artery in-stent restenosis: evidence from an updated meta-analysis. Eur Radiol 28:1373–1382. 10.1007/s00330-017-5097-0 29124384 10.1007/s00330-017-5097-0

[44] Williams MC, Kwiecinski J, Doris M, et al (2021) Sex-specific computed tomography coronary plaque characterization and risk of myocardial infarction. JACC Cardiovasc Imaging 14:1804–1814. 10.1016/j.jcmg.2021.03.004 33865779 10.1016/j.jcmg.2021.03.004 PMC8435010

[45] Park D-W, Kim H, Singh A, Brown DL (2024) Prophylactic stenting of vulnerable plaques: pros and cons. EuroIntervention 20:e278–e280. 10.4244/EIJ-E-24-00004 38436373 10.4244/EIJ-E-24-00004 PMC10905194

